# Endoscopic Removal of an Inadvertently Swallowed Toothbrush in the Emergency Department

**DOI:** 10.1155/2012/568163

**Published:** 2012-07-02

**Authors:** Martyn Harvey, Grant Cave, Gaynor Prince

**Affiliations:** ^1^Department of Emergency Medicine, Waikato Hospital, Pembroke Street, Hamilton, New Zealand; ^2^Department of Emergency Medicine, Hutt Hospital, High Street, Lower Hutt, Wellington, New Zealand

## Abstract

A 16-year-old girl inadvertently swallowed a toothbrush during attempted manual induction of emesis. The 20 cm toothbrush was successfully removed via overtube facilitated endoscopy using a retractable snare while the patient was sedated in the emergency department.

## 1. Introduction

Foreign body ingestion represents a common emergency department presentation. While the vast majority of ingested foreign bodies traverse the gastrointestinal tract with nil adverse sequelae [1, 2], few require early endoscopic removal due to their corrosive nature [3], potential for alimentary tract perforation [4], or physical size [4]. We report a 16-year-old girl who accidentally swallowed a toothbrush which was extracted via endoscopy without complication.

## 2. Case

A 16-year-old female presented to our tertiary care facility two hours following ingestion of a household toothbrush. She admitted to excess alcohol intake the evening prior and have woken feeling “hung-over.” Due to ongoing nausea, she had attempted to induce emesis via manual pharyngeal stimulation with a toothbrush. However, during this process, she claimed to have “choked,” lost control of the toothbrush handle, and ingested the “white-and-orange” toothbrush whole. 

Clinical examination revealed a well-appearing 16-year-old female in no distress. Mild pharyngeal abrasions were apparent on examination of the oral cavity. Cardiovascular, respiratory, and abdominal examination proved unremarkable; in particular no abdominal tenderness was reported on manual palpation. Soft tissue lateral X-ray of the neck and chest X-ray revealed no evidence of ingested foreign body. 

**Figure 1:**
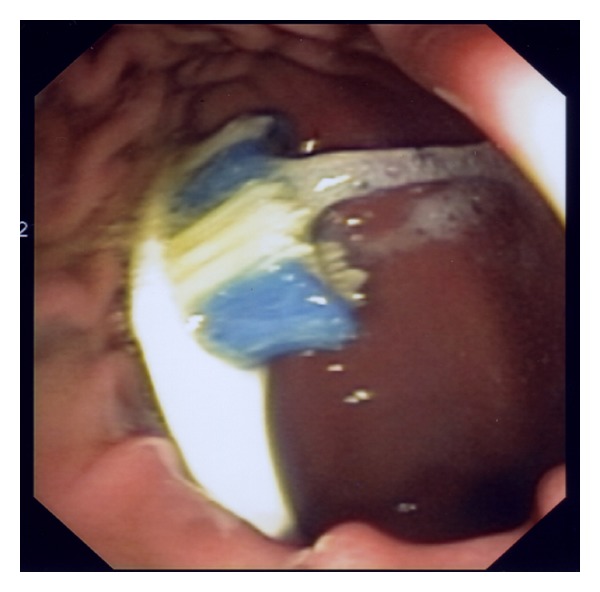
Endoscopic view of toothbrush in gastric antrum, handle having disappeared into the pylorus.

**Figure 2:**
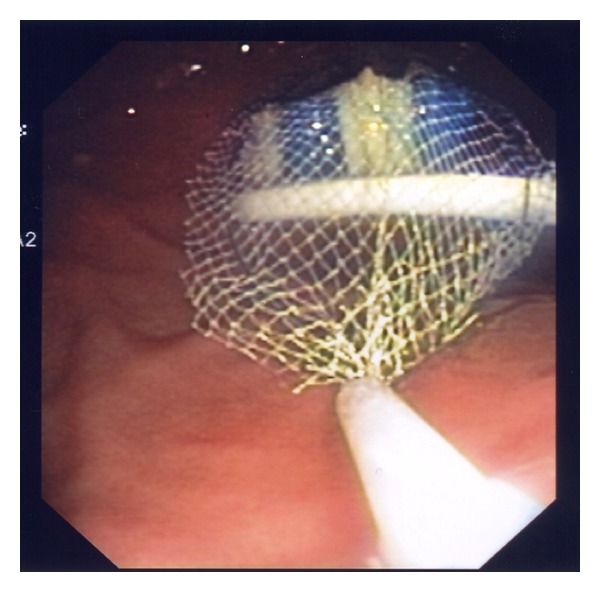
Attempted snare of toothbrush.

**Figure 3:**
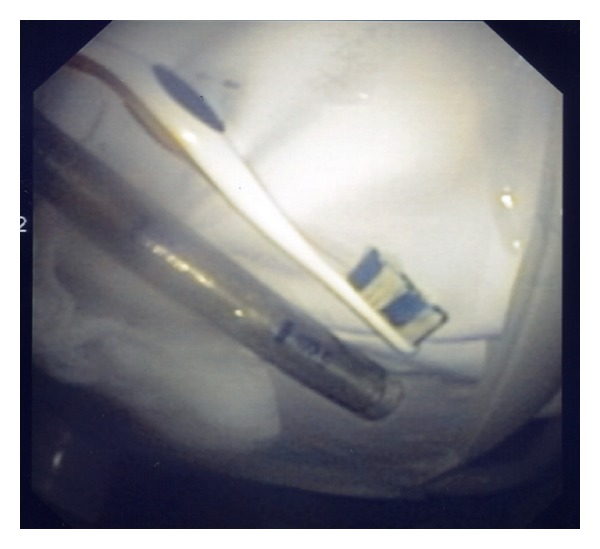
Extracted toothbrush and endoscopic overtube.

Endoscopic retrieval of the toothbrush was undertaken in the emergency department. Given the dimension of the ingested foreign body and anticipated requirement for overtube utilization, airway protection was afforded with endotracheal intubation following standard rapid sequence intubation (RSI). Sedation was maintained with propofol/ketamine combination. Gastroscopy was performed with visualization of the distal (brush) end of toothbrush protruding from the gastric outlet, the proximal (handle) having already traversed the pylorus (Figure 1). The brush was snared (Figure 2), and withdrawn en masse with the pictured overtube (Figure 3) to the level of cricopharyngeus. Removal was then completed under direct laryngoscopy with Magills forceps. Relook endoscopy was undertaken with confirmation of nil-induced trauma.

The patient was extubated uneventfully and discharged two hours later in possession of the offending toothbrush. Future manual pharyngeal stimulation for the purposes of inducing emesis was discouraged.

## 3. Discussion

Inadvertent or intentional ingestion of foreign objects frequently results in emergency department attendance [2]. In the paediatric population, ingestion of foreign bodies is most commonly accidental and associated with oral exploration of the environment. In adults, aetiologic factors associated with foreign body ingestion include poor visual acuity, alcohol and drug addiction, dental prostheses, and psychiatric disease [5]. Management of ingested foreign objects varies depending on the nature of the ingested object, its location at presentation, and patient factors. While the vast majority of foreign bodies that reach the stomach will traverse the alimentary tract spontaneously [1, 2, 6], requiring little more than observation in an outpatient setting, few require targeted endoscopic or surgical removal to prevent complications of impaction, obstruction, or perforation leading to abscess formation, fistulae, or generalized peritonitis.

Oesophageal foreign bodies as a group require early intervention (within 24 hours) because of their potential to induce respiratory complication, oesophageal erosions, or, in the most extreme instances, aortooesophageal fistula [7]. Ingested button batteries that become lodged in the oesophagus require still greater expedition of removal given the risk of corrosive tissue damage [3]. Sharp foreign bodies likewise require endoscopic removal due to the increase risk of gastrointestinal perforation (15–35% [2]). Recent recognition of the dangers posed by multiple ingested magnets, due to pressure necrosis of bowel wall caught between adjacent magnets, requires vigilance and consideration of early endoscopic removal before small bowel transit [8].

Swallowed linear foreign bodies of greater than 6–10 cm length, such as toothbrushes, present a unique problem in that, given their length, they are unable to negotiate the curvature of the duodenum with its fixed retroperitoneal attachment [9]. Despite greater than forty literature reports of toothbrush ingestion, none exist of spontaneous whole bowel transit. Prolonged lodgment in the duodenum may result in pressure necrosis and subsequent gastrointestinal perforation. Endoscopic removal of ingested toothbrushes is therefore recommended at the earliest possible juncture, with laparoscopic gastrostomy endorsed for failed endoscopic retrieval [9, 10].

## 4. Conclusion

Toothbrush ingestion, while uncommon, presents a unique clinical challenge due to the inability of this foreign body to undergo gastrointestinal passage, spontaneously. Early endoscopic retrieval is therefore recommended to reduce morbidity associated with prolonged impaction.
